# Supplementary material for: Causal Relationship between Aging and Anorexia Nervosa: A White-Matter-Microstructure-Mediated Mendelian Randomization Analysis
Source: Biomedicines. 2024 Aug 16;12(8):1874. doi: 10.3390/biomedicines12081874 (PMC11351342; doi:10.3390/biomedicines12081874)
Supplement: Supplementary file 1 [file biomedicines-12-01874-s001.zip › Supplementary Figure/Figure S4/Singe SNP plot of MR analysis for TL to ubm-b-1676.pdf]

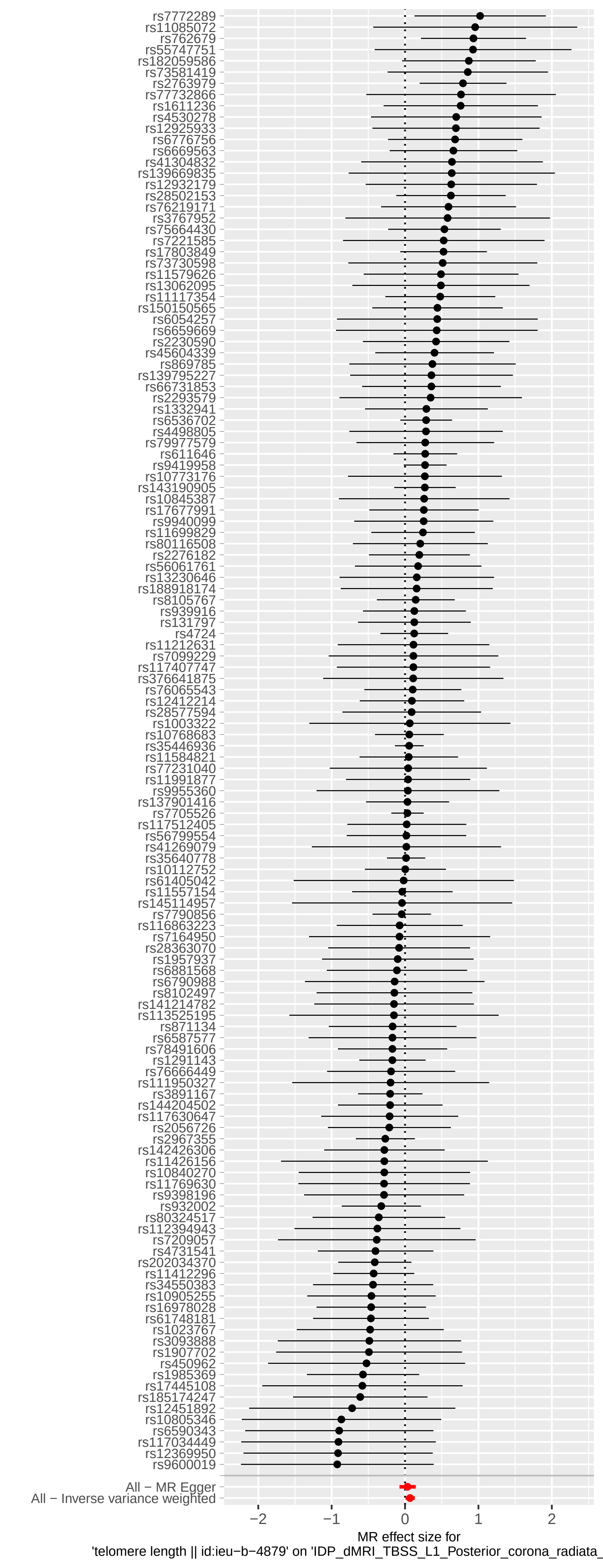
rs7772289
rs11085072
rs762679
rs55747751
rs182059586
rs73581419
rs2763979
rs77732866
rs1611236
rs4530278
rs12925933
rs6776756
rs6669563
rs41304832
rs139669835
rs12932179
rs28502153
rs76219171
rs3767952
rs75664430
rs7221585
rs17803849
rs73730598
rs11579626
rs13062095
rs11117354
rs150150565
rs6054257
rs6659669
rs2230590
rs45604339
rs869785
rs139795227
rs66731853
rs2293579
rs1332941
rs6536702
rs4498805
rs79977579
rs611646
rs9419958
rs10773176
rs143190905
rs10845387
rs17677991
rs9940099
rs11699829
rs80116508
rs2276182
rs56061761
rs13230646
rs188918174
rs8105767
rs939916
rs131797
rs4724
rs11212631
rs7099229
rs117407747
rs376641875
rs76065543
rs12412214
rs28577594
rs1003322
rs10768683
rs35446936
rs11584821
rs77231040
rs11991877
rs9955360
rs137901416
rs7705526
rs117512405
rs56799554
rs41269079
rs35640778
rs10112752
rs61405042
rs11557154
rs145114957
rs7790856
rs116863223
rs7164950
rs28363070
rs1957937
rs6881568
rs6790988
rs8102497
rs141214782
rs113525195
rs871134
rs6587577
rs78491606
rs1291143
rs76666449
rs111950327
rs3891167
rs144204502
rs117630647
rs2056726
rs2967355
rs142426306
rs11426156
rs10840270
rs11769630
rs9398196
rs932002
rs80324517
rs112394943
rs7209057
rs4731541
rs202034370
rs11412296
rs34550383
rs10905255
rs16978028
rs61748181
rs1023767
rs3093888
rs1907702
rs450962
rs1985369
rs17445108
rs185174247
rs12451892
rs10805346
rs6590343
rs117034449
rs12369950
rs9600019
All − MR Egger
All − Inverse variance weighted
−2
−1
0
1
2
MR effect size for
'telomere length || id:ieu−b−4879' on 'IDP_dMRI_TBSS_L1_Posterior_corona_radiata_
